# Perinatal nutrition programs neuroimmune function long-term: mechanisms and implications

**DOI:** 10.3389/fnins.2013.00144

**Published:** 2013-08-12

**Authors:** Sarah J. Spencer

**Affiliations:** School of Health Sciences and Health Innovations Research Institute, RMIT UniversityMelbourne, VIC, Australia

**Keywords:** hypothalamic-pituitary-adrenal axis, obesity, glucorticoids, gut microbiota, epigenetics

## Abstract

Our early life nutritional environment can influence several aspects of physiology, including our propensity to become obese. There is now evidence to suggest perinatal diet can also independently influence development of our innate immune system. This review will address three not-necessarily-exclusive mechanisms by which perinatal nutrition can program neuroimmune function long-term: by predisposing the individual to obesity, by altering the gut microbiota, and by inducing epigenetic modifications that alter gene transcription throughout life.

## Perinatal dietary influence on immune system development

The immune system of a newborn animal is relatively naïve and influences from the environment are necessary to allow it to become fully functional. Early exposure to pathogens develops an adaptive (Flajnik and Kasahara, 2010) and innate (Galic et al., 2009; Spencer et al., 2011) immunity that will facilitate appropriate responses to additional pathogens throughout life. However, there is now evidence that the early life diet is also crucial in programming long-term immune function.

### Specific nutrients in perinatal diet influence immune system development

Specific nutrients within an individual diet can influence immune system development in different ways. Thus, antioxidants, oligosaccharides, polyunsaturated fatty acids (PUFAs), folate, and other vitamins have all been implicated in programming the developing immune system (West et al., 2010). For example, omega-3 PUFAs, those found in fish, fish oils, green plants, and some nuts and seeds (Huffman et al., 2011; Kremmyda et al., 2011), have an anti-inflammatory role. They inhibit cytokine production and may also alter gene expression and stimulate eicosanoid metabolism to control inflammation (Shek et al., 2012). Thus, a diet high in omega-3 PUFAs leads to a suppression of arachidonic acid-derived eicosanoids such as prostaglandin E2 (PGE2). PGE2 exerts pro-inflammatory effects and reduces the production of T helper type 1 (Th1) cytokines, such as interferon (IF) γ and interleukin (IL)2 and enhances production of Th2 cytokines like IL-4 and IL-5. A shift in the Th1/Th2 balance toward a Th2-dominant profile is associated with impaired immune tolerance and allergies (Gottrand, 2008). High maternal intake of fish is thus associated with protection, in the infant, from allergic diseases such as eczema and asthma (Calvani et al., 2006; Romieu et al., 2007; Sausenthaler et al., 2007). Omega-6 PUFAs, those found in vegetable oils (Huffman et al., 2011; Kremmyda et al., 2011), are pro-inflammatory and may contribute to metabolic syndrome and cardiovascular disease (Patterson et al., 2012). A shift in the ratio of omega-3 to omega-6 PUFA intake in the diet to favor omega-6 encourages an allergenic Th2-dominant profile and is thus likely contributing to the recent increase in the incidence of childhood allergies (Shek et al., 2012). Folic acid is another example of a dietary component that influences immune development. Folic acid supplementation during pregnancy reduces the risk of neural tube defects and other congenital malformations (Wilcox et al., 2007), but it also predisposes infants toward allergies and immune dysfunction. For instance, folate supplementation in the first trimester of pregnancy predisposes infants to developing wheeze and respiratory infections in early life (Haberg et al., 2009).

### Perinatal diet influences immune system development through the gut microbiota

One mechanism by which specific nutrients in the early life diet may be able to influence the adult immune system is by affecting the development, diversity, and function of the gut microbiota. In humans, the gastrointestinal tract is home to more than 100 trillion bacteria comprised of more than 1000 species (Qin et al., 2010). It also hosts numerous viruses, archaea, parasites, and fungi that together make up the gut microbiota (Ashida et al., 2012). This microbiota exists in a symbiotic relationship with its human hosts and can influence barrier function, trophic effects, metabolism, and the development of the adaptive and innate immune systems (Matamoros et al., 2013).

An adult's gut microbiome can be influenced by long-term changes in environmental factors. Hildebrandt and colleagues have shown 3 months of high fat diet (HFD)-feeding can influence a change in gut microbiota composition toward an increase in *Firmicutes* and *Proteobacteria* and a decrease in *Bacteroidetes* phyla in female mice (Hildebrandt et al., 2009). Although these changes were independent of obesity, other groups have shown a high fat, high sugar diet encourages increased adiposity and this phenotype can be transmitted to initially lean (normal diet) animals via transplantation of the microbiota (Turnbaugh et al., 2008). Most evidence, however, suggests the adult gut flora is very stable and short-term environmental influences in adulthood have limited effect (Wu et al., 2011). An infant, on the other hand, is not born with an established gut microbiome. Rather, the gut is colonized from bacteria in the environment in the first hours to days of life and the microbiome gains diversity and becomes stable and adult-like by around 3 years of age (Mackie et al., 1999; Palmer et al., 2007). In particular, an individual's diet during the early colonization phase can be tremendously important in determining the later composition of the gut microbiota.

Breast milk is a major source of the bacteria that colonize the gut (Martin et al., 2012). Breast-fed infants have higher counts of *Bifidobacteria*, *Lactobacilli*, and lower counts of *Bacteroides, Clostridium coccoides* group, *Staphylococcus*, and *Enterobacteriaceae* than formula-fed (Rinne et al., 2005; Fallani et al., 2010). Breast milk is also rich in oligosaccharides, which have a strong pre-biotic effect, promoting bacterial growth (Sela and Mills, 2010). Oligosaccharides found in human milk can improve the diversity of the microbiota, particularly promoting growth and metabolism of *Bifidobacteria* (Scholz-Ahrens et al., 2007). Human milk oligosaccharides can even improve glucose homeostasis (Laitinen et al., 2009). This interaction between breast milk oligosaccharides and gut bacteria also encourages immune system development and prevention of disease (Innis, 2007). Maternal diet strongly influences the composition of the breast milk and probably, therefore, the types of bacteria available to colonize the infant's gut. For instance, in rats, a maternal diet high in olive oil leads to high oleic-acid levels in the milk. A maternal diet high in PUFA is reflected in high PUFA concentrations in milk. Saturated fats are also transferred to the milk (Priego et al., 2013). When infants are introduced to a more complex diet at weaning there is a marked increase in *Bacteroidetes*, and a shift toward a more diverse colony (Koenig et al., 2011). The infant's post-weaning diet can therefore also affect the makeup of the gut microbiota. High dietary fat, for example, can cause a shift toward increased representation of *Clostridium populeti* bacteria and a reduction in *Lactobacillus* and *Bacteroides* species (Patrone et al., 2012). In weaned piglets the quantity and type of carbohydrate in the diet can influence the gut microbiota so that a diet high in hulled barley supplemented with beta-glucan encourages a *Lactobacilli*-dominant gut microbiota (Pieper et al., 2008).

Exactly what this means for the infant is yet unclear as there is no consensus as to what constitutes a “normal” gut microbiome (Matamoros et al., 2013). However, changing the makeup of the bacterial colony can certainly influence the immune system long-term. Infants given *Bacteroides fragilis* supplements early in life have high salivary secretory immunoglobulin A (IgA) and more basal IFN-γ production. They also have reduced expression of the pathogen-associated molecular pattern receptor, toll-like receptor (TLR)4, mRNA and an attenuated pro-inflammatory response to stimulation with lipopolysaccharide (LPS) than those not given the *B. fragilis* supplements (Gronlund et al., 2000; Sjogren et al., 2009). These findings suggest the possibility that early colonization with *B. fragilis* can accelerate the maturation of the IgA system, leading to improved Th1/Th2 balance, a reduced likelihood of allergies developing, and a reduced response to LPS. A *Lactobacilli*-dominant colonization in infancy also reduces the likelihood the child will develop allergies by age five, while a *Staphylococcus aureaus*-dominant colonization has the opposite effect (Johansson et al., 2011). This latter bacterium has been linked to asthma and allergic rhinitis in childhood (Bjorksten et al., 2001). *Lactobacillis* tends to suppress numbers of interleukin (IL)-4, IL-10, and IFN-γ -secreting cells after stimulation with phytohaemagglutinin, whereas early life *S. aureaus* colonization has the opposite effect. Colonization with *Lactobacilli* also lowers cytokine responses to stimulation with an allergen (Martino et al., 2008). The *Lactobacilli* data suggest that this bacterium is able to suppress the immune response.

### Perinatal diet influences immune system development through epigenetic modifications

An interrelated mechanism by which perinatal diet can influence the innate immune system is through changes in the epigenome. Epigenetics refers to stable, heritable, environmentally-induced modifications to gene expression that occur independently of alterations to the DNA sequence (Christensen and Marsit, 2011). These modifications include changes in cytosine methylation, histone modification, and changes in non-coding RNAs such as microRNAs (Milagro et al., 2013). Together these mechanisms are responsible for regulating the degree of expression of a particular gene and the timing of its expression (Zeisel, 2009; McKay and Mathers, 2011). While epigenetic research is still a very young field, there is a large body of evidence accumulating to suggest diet, particularly in early life, can influence this epigenome long-term (Lillycrop et al., 2008). There are now data revealing almost every dietary component, from broccoli to betaine can influence the epigenome. Broccoli, for example contains sulforaphane, which induces histone modifications and has been implicated in preventing cancer (Dashwood and Ho, 2008; Delage and Dashwood, 2008; Nian et al., 2009). Betaine is found in grains and some vegetables and has been found to influence DNA methylation to promote fetal brain development (Sinclair et al., 2007; Mehedint et al., 2010; Zeisel, 2011).

**Figure 1:**
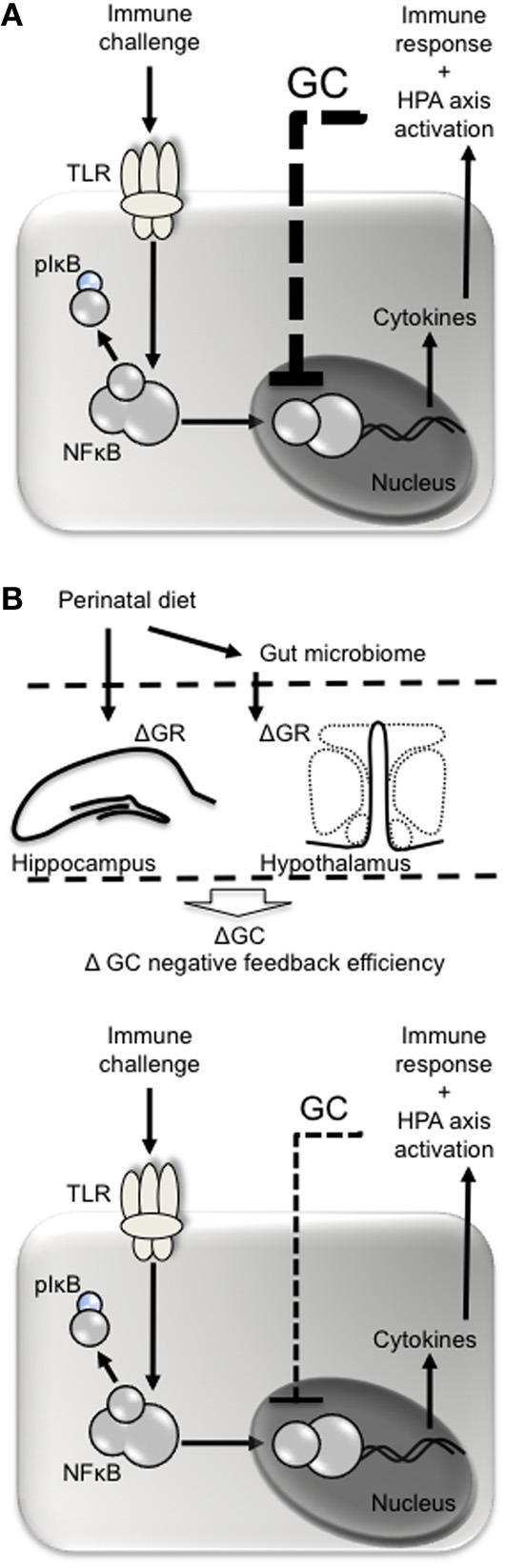
**Perinatal diet may influence glucocorticoid negative feedback following immune challenge. (A)** Pathogens such as lipopolysaccharide act at toll-like receptors (e.g., TLR4) on immune cells leading to phosphorylation of inhibitory factor (I)κ B, releasing nuclear factor (NF)κ B from its complex and allowing it to be translocated to the nucleus. NKκ B is responsible for the transcription of pro- and anti-inflammatory cytokines, the former of which stimulate the cyclo-oxygenase 2-mediated conversion of aracidonic acid into prostaglandins. Prostaglandins (e.g., PGE2) act at the brain to stimulate fever and sickness behavior and recruit the HPA axis. Once released, glucocorticoids (GC) negatively feed back to inhibit further NFκ B-mediated transcription of cytokines. **(B)** The perinatal diet may influence glucocorticoid negative feedback by altering expression of glucocorticoid receptors (GR) in the hippocampus and hypothalamus leading to less efficient glucocorticoid-mediated inhibition of NFκ B and an exacerbated immune response.

Dietary components may also alter the epigenome to influence immune function, and there is a particular window of vulnerability for this during early development (West et al., 2011). Folate for example is a methyl donor. At least in mice, folate supplementation in pregnancy causes DNA hypermethylation, particularly in key metabolic genes (Waterland et al., 2008). Supplementation with methyl donors such as folate is also associated with altered immune function resulting in increased development of allergic asthma and eczema (Hollingsworth et al., 2008; Haberg et al., 2009). PUFAs also have the potential to cause epigenetic modifications. Crucially, PUFAs may be able to alter nuclear factor κ B (NFκ B)-mediated transcription of pro-inflammatory cytokines to influence the sensitivity of the immune response (Benatti et al., 2004; Waterland, 2006). NFκ B is a transcription factor responsible for regulating the expression of more than 400 genes, including those responsible for pro-inflammatory cytokines, chemokines, and adhesion molecules (Vanden Berghe et al., 2006). NFκ B-mediated transcription may be particularly vulnerable to early life influence and may be a principal mechanism by which epigenetic programming can influence immune system development long-term (Benatti et al., 2004; Vanden Berghe et al., 2006). NFκ B itself is closely regulated by glucocorticoids and these are influenced by hypothalamic-pituitary-adrenal (HPA) axis activation (Sapolsky et al., 2000). Epigenetic programming by early life stress can result in changes in the methylation status of the glucocortiocoid receptor (GR) in the hippocampus and hypothalamus, altering negative feedback onto the hypothalamus, and thus HPA axis sensitivity (Liu et al., 1997; Weaver et al., 2004; Stevens et al., 2010; Begum et al., 2012). Early life diet can also impact HPA axis reactivity long-term (Boullu-Ciocca et al., 2005; Spencer and Tilbrook, 2009; Bulfin et al., 2011), altering the glucocorticoid response to stress. Enhanced circulating glucocorticoid levels in response to stress then feed back to inhibit NFκ B-mediated cytokine production (Figure 1). The HPA axis in particular will be discussed in the next section.

## Perinatal dietary influence on adiposity—links to immune system development

Dietary factors in early life clearly have a crucial influence on immune system development. The second half of this review will focus on how early life nutrition can program a pro-inflammatory basal immune profile by pre-disposing an individual to an obese phenotype.

Obesity is becoming a huge problem worldwide. In developed countries such as Australia and the US, 70–74% of adult males and 56–64% of adult females are now either overweight or obese, with 28% of both classified as obese (BMI > 30). As many as 25–32% of Australian and US children are classified as overweight or obese (Cretikos et al., 2008; Nhanes, 2009-2010; AHS, 2011-2012).

### Perinatal nutrition can program adult weight and metabolism leading to adipose-dependent changes in immune function

Obesity itself, whether due to metabolic changes programmed in early life or to adult factors, is linked to changes in the inflammatory profile. It is now recognized that obesity is associated with, and may even be precipitated by, a chronic low-grade systemic and local inflammation (Gregor and Hotamisligil, 2011). This metabolic inflammation can contribute to insulin- and leptin-resistance at various levels, including at the hypothalamus (Thaler and Schwartz, 2010).

Dietary factors such as PUFAs and glucose, as well as changes in the gut microbiota, are able to trigger a chronic low-grade inflammatory profile initially in white adipose tissue (WAT). This change is characterized by macrophage infiltration into WAT, apoptosis and necrosis of adipocytes, and reduced vascularity (Shu et al., 2012). These changes result in an abnormal preponderance of adipose-tissue macrophages, and these can make up almost 40% of the cells in obese adipose tissue (Weisberg et al., 2003; Xu et al., 2003). Adipose tissue macrophages, and potentially an increase in pattern recognition receptors on adipocytes themselves, lead to local inflammation with a predominance of pro-inflammatory over anti-inflammatory cytokines released (Shu et al., 2012). Hotamisligil and colleagues showed early on there is a substantial increase in expression of the pro-inflammatory cytokine tumor necrosis factor (TNF)α in several rodent models of obesity and that neutralizing TNFα could improve insulin-sensitivity in these animals (Hotamisligil et al., 1993, 1995; Uysal et al., 1997). The pro-inflammatory profile in the adipose leads to cytokine, adipokine, and fatty acid release into circulation, which have downstream effects on liver, muscle, and brain, and ultimately contribute to insulin-resistance (Shu et al., 2012).

As a result of these changes in the inflammatory profile, obese subjects have compromised immune function and are more likely to die from an acute infection than those of normal weight (Falagas and Kompoti, 2006). For instance, excessive body weight gain immediately postnatally predisposes infants to atopy and wheezing disorders (Pike et al., 2010). Obese patients in general are also twice as likely to die in intensive care due to infection-related complications as normal weight patients (Falagas and Kompoti, 2006).

It has been clear for some time that early life nutrition is able to program growth and can influence development of the central pathways subserving feeding and metabolism (Spencer, 2012). Babies born to overweight or obese mothers are significantly more likely to become overweight or obese themselves (Dabelea et al., 2000; Ruager-Martin et al., 2010), and babies born to mothers who ate a high fat, junk food diet while pregnant have higher levels of body fat when they are born, irrespective of whether or not the mothers were obese during pregnancy (Albuquerque et al., 2006; Srinivasan et al., 2006; Ashino et al., 2012). Associated with this excess body fat are indices of metabolic syndrome such as hyperinsulinemia and insulin resistance (Dabelea et al., 2000; Boney et al., 2005; Sewell et al., 2006; Catalano et al., 2009).

Paradoxically, babies that were undernourished *in utero* are also more likely to develop obesity and associated metabolic disorders (Spencer, 2012). In the first instance, *in utero* factors that cause the baby to be born small may also alter its metabolic pathways to encourage energy storage when food is available (Vickers et al., 2000, 2003; Bellinger et al., 2004; Bellinger and Langley-Evans, 2005). Secondly, preferred practise with small for gestational age babies is a program of intensive feeding to encourage appropriate brain and lung development (Lubchenco et al., 1972a,b; Brandt et al., 2003) and this catch up growth in the postnatal period also predisposes an individual to obesity (Ong et al., 2000, 2006; Brandt et al., 2003; Desai et al., 2005).

The importance of these findings is reflected in statistics showing overweight children are significantly more likely to be overweight adults than those of normal weight. As mentioned, excessive weight gain in the first week of life increases the long-term risk of obesity (Stettler et al., 2005). Furthermore, compared with children with a BMI below the 50th percentile, children between the 50 and 74th percentiles of BMI are approximately five times more likely to become overweight adults (Baird et al., 2005; Field et al., 2005; Druet et al., 2012).

### Perinatal nutrition can program changes in immune function that are independent of adiposity

We can conclude from these studies there is an obvious connection between early life events programming an increased propensity to obesity and obesity itself resulting in a basal pro-inflammatory profile and susceptibility to infection. However, it is also apparent being overweight in early life can have independent and compounding effects on the inflammatory profile in adulthood.

Interesting evidence for the long-term effects of early life diet on the adult immune system comes from individuals who were undernourished *in utero* or as infants and did not develop obesity. Thus, a study of three rural villages in Gambia revealed subjects were significantly more likely to die of infectious disease in adulthood if they had been born during the nutritionally debilitating “hungry” season of July–December than during January–June when food was plentiful (Moore et al., 1999). A calorie restricted perinatal diet has also been shown to influence macrophage activation in adulthood so that adult rats undernourished during lactation had fewer alveolar macrophages and these released less nitric oxide in response to a fluoxetine challenge (Ferreira et al., 2009). Similarly, adult rats undernourished during lactation showed no change in immune parameters after an immune challenge either under control conditions or after being subjected to footshock, while control rats (normal diet during lactation) had elevated leukocyte counts and antibody titers (Barreto-Medeiros et al., 2007). These data suggest neonatal malnutrition can lead to a less reactive or less efficient immune response.

There is also some evidence that animals made obese as a result of perinatal diet can have changes in neuroimmune function in later life that are independent of the obesity *per se*. Several groups have now shown rats suckled in small litters, where they have greater access to the dam's milk, gain weight faster and maintain a higher body weight into adulthood (Plagemann et al., 1999; Schmidt et al., 2001; Morris et al., 2005; Rodel et al., 2008). We have shown these overweight rats, both males and females, have a significantly exacerbated neuroimmune response to LPS. This response is categorized by exacerbated NFκ B activation in the overweight rats, more circulating pro-inflammatory cytokines, and bigger fevers (Clarke et al., 2012).

Importantly, there are some fundamental differences between the changes in neuroimmune function in rats made overweight due to early life overfeeding and those in rats made overweight due to HFD-feeding in adulthood. Firstly, neonatally overfed rats do not have a profile of basal inflammation. There are no differences in basal circulating pro-inflammatory cytokine concentrations between those suckled in small litters (overweight) and those suckled in control litters (Clarke et al., 2012). As discussed above, several studies have shown human (Hak et al., 1999; Yudkin et al., 1999) and rodent (Hotamisligil et al., 1993) obese subjects have higher levels of circulating pro-inflammatory cytokines under unstimulated conditions, reflecting a pro-inflammatory profile. This difference may be a result of the degree of obesity, dietary composition, and/or that the perinatal overfeeding is able to prime the system to display an over-active response to immune challenge without affecting the basal inflammatory profile (Pohl et al., 2009).

The second key difference between immune dysfunction as a result of perinatal obesity and that of diet-induced obesity in adulthood is that perinatal obesity leads to an exacerbated immune response to a TLR4-mediated challenge, but not to a TLR3-mediated one (Clarke et al., 2012). The TLR family contains as many as 13 mammalian TLR, most of which respond to specific pathogen-associated molecular patterns. In the case of Gram negative bacteria, the pyrogenic moiety, LPS, interacts with cluster of differentiation (CD)14 on the cell membrane, allowing MD2 to associate with TLR4. This interaction activates a myeloid differentiation primary response gene (MyD88)-dependent pathway, culminating in the phosphorylation of NFκ B-interacting inhibitory factor (I) κ B, which releases NFκ B from its complex. NFκ B is then translocated to the nucleus of the cell where it stimulates the transcription of pro- and anti-inflammatory cytokines (Cartmell et al., 2003; Conti et al., 2004; Galic et al., 2009). Pro-inflammatory cytokines act at the brain to stimulate cyclo-oxygenase 2-mediated conversion of arachadonic acid into prostaglandins. These then act in the ventromedial preoptic area of the hypothalamus to disinhibit neuronal pathways that normally stimulate heat conservation, ultimately resulting in a regulated increase in body temperature; fever (Figure 1A) (Blatteis et al., 2000; Morrison et al., 2008). In the case of a virus, the viral double-stranded RNA interacts with TLR3 to stimulate the immune cascade via an interferon regulatory factor 3-dependent pathway. Polyinosinic:polycytidylic acid (PolyI:C) is a synthetic double-stranded RNA that mimics a virally-induced immune response and fever by activating TLR3. In neonatally overfed rats the response to LPS is exacerbated, while the response to PolyI:C remains normal (Clarke et al., 2012).

As with TLR4, there is increased TLR3 expression in neonatally overfed rat adipose tissue (Clarke et al., 2012). However, unlike in humans with adult-onset obesity (MMWR, 2009; Fuhrman et al., 2011) and adult HFD-fed rodents (Smith et al., 2007), the immune response to a TLR3 ligand is not altered in rats made obese due to neonatal overfeeding (Clarke et al., 2012). A possible explanation for differential effects on TLR4 and TLR3 signaling is in the receptor location, with TLR4 being membrane-bound and TLR3 internalized (Kumar et al., 2009; Konner and Bruning, 2011). Thus, although obesity in general may increase TLR3 expression, perinatally-induced obesity may not cause corresponding changes in transport of the ligand into the cell. For the patient, this may mean early-life programming of obesity may be associated with some form of protection against a viral infection in comparison with adult-onset obesity.

Perinatal overfeeding is, unlike adult onset obesity, also able to exacerbate immune responses independently of sickness behavior. Generally, an immune response elicits a variety of sickness behaviors in addition to the pro-inflammatory and febrile changes. These include anorexia, lethargy, depression, reduced activity, loss of libido (Dantzer and Kelley, 2007). Although there is a typical expression of sickness behavior with LPS in perinatally overfed rats, this is not exacerbated in these animals as the pro-inflammatory and febrile responses are (Clarke et al., 2012). In contrast, adult-onset obesity is strongly associated with an increase in sickness behavior relative to lean adults (Lawrence et al., 2012). Several aspects of sickness behavior are likely to be mediated centrally. For instance, leptin is a significant modulator of the anorexia associated with infection (Luheshi et al., 1999), and treatment with leptin anti-serum can reverse LPS-induced anorexia (Sachot et al., 2004; Harden et al., 2006). Leptin responses to LPS are similar in neonatally overfed and control rats despite pronounced differences in other cytokines, potentially facilitating similar sickness responses (Clarke et al., 2012).

It is yet unclear what this absence of an exacerbated sickness response after perinatal overfeeding would mean for a human subject. On one hand the subject is likely to be resilient to the feeling of sickness associated with an immune challenge, despite having exacerbated pro-inflammatory and febrile response, allowing them to continue life as normal when sick. On the other hand, sickness behavior is very important in promoting withdrawal so the body's resources are fully available to effectively combat the infection (Carlton et al., 2012).

### The hypothalamic-pituitary-adrenal axis

As a possible key explanation for how the early life nutritional environment apparently programs adult immune function independently of obesity is in epigenetic changes to key aspects of the HPA axis. The HPA axis plays a significant modulatory role in the immune response with glucocorticoids acting to inhibit NFκ B activation and downstream transcription of pro- and anti-inflammatory cytokines (Figure 1) (Spencer et al., 2011). The HPA axis is also exceptionally sensitive to the early life environment. It has previously been established early life changes in HPA axis function are associated with changes in responses to LPS in adulthood. For instance, early life exposure to an immune challenge can permanently alter HPA axis function (Shanks et al., 1995, 2000; Hodgson et al., 2001). Early life immune challenge leads to an exacerbated HPA axis response to LPS in later life and blocking this increase with RU486 can restore a normal febrile response and cytokine profile (Ellis et al., 2005; Mouihate et al., 2010). At least some of the changes in HPA axis function that derive from early life events are linked to epigenetic modifications. For instance, rats that received high levels of care from their dams as pups (high levels of licking and grooming) have hypomethylation of the GR in the hippocampus and this is associated with increased hippocampal GR mRNA and a more efficient glucocorticoid negative feedback response to stress compared with rats that were given less attention as pups (Liu et al., 1997; Weaver et al., 2004). The hippocampal GR system plays a crucial role in glucocorticoid negative feedback regulation of the HPA axis, with glucocorticoids acting on GR at the hippocampus to inhibit PVN activation (De Kloet et al., 2009). As such, epigenetic modification of hippocampal GR may have significant effects on HPA axis function (Liu et al., 1997; Weaver et al., 2004; Mueller and Bale, 2006). Undoubtedly, glucocorticoid negative feedback at the hypothalamus itself is also important and can be altered by changes to the epigenome. For instance, maternal undernutrition is linked to increased histone acetylation and hypomethylation of the GR in the hypothalamus of the offspring, with a substantial increase in GR expression in this region. These modifications are closely linked with enhanced weight gain, and subsequent obesity, in these offspring (Stevens et al., 2010; Begum et al., 2012).

Early life diet is certainly capable of altering how the HPA axis functions. Females that become overweight as a result of early life overfeeding have enhanced PVN and corticosterone responses to acute stress (Spencer and Tilbrook, 2009). Conversely, males made lean by early underfeeding have more efficient HPA axis responses to stress, with reduced PVN neuronal activation and corticosterone responses that return to baseline more quickly (Bulfin et al., 2011). Neonatally overfed rats also have increased expression of the GR and increased glucocorticoid signaling in adipose tissue as adults (Boullu-Ciocca et al., 2005). In neonatally overfed rats, adult LPS leads to a significantly enhanced PVN response to stress and a corticosterone response that is significantly less efficient. Plasma corticosterone reaches a peak 30 min after LPS in control animals before returning to baseline, but in neonatally overfed rats the corticosterone levels still appears to be increasing after 90 min (Clarke et al., 2012). Together these data indicate early life overfeeding may lead to impaired development of central and peripheral HPA axis and glucocorticoid regulation resulting in altered HPA axis function in later life. These changes are likely to be responsible for a delay in the glucocorticoid response to an immune challenge, which would culminate in exacerbated PVN/HPA axis activation and a less effective glucocorticoid-mediated suppression of cytokine release, and fever (Figure 1).

### Sexual dimorphism in perinatal nutritional programming of immune function

Obesity can be manifested very differently in males and females. For instance, Australian and US statistics show a greater proportion of males than females are overweight or obese (Cretikos et al., 2008; Nhanes, 2009-2010; AHS, 2011-2012). Males are also more likely to accumulate visceral fat, a distribution that is more closely associated with complications such as heart disease (Bjorntorp, 1996). HPA axis responses to psychological stress also differ as a function of adiposity between males and females. Thus, perceived stress has been associated with greater increases in BMI in women, but not in men (Fowler-Brown et al., 2009), and female rats overfed as neonates have exacerbated HPA axis responses to restraint, while males do not (Spencer and Tilbrook, 2009). On the other hand, there is little evidence to suggest there are substantial sex differences in the neuroimmune response to an immune challenge in terms of how it is programmed by the perinatal environment. Most studies examining the effects of changes in gut flora on immune function have either only included males, or have found no effect of sex on immune-related outcomes (e.g., Calvani et al., 2006; Haberg et al., 2009; Patterson et al., 2012; Shek et al., 2012). There are some sex differences in the effects of gut flora on central nervous system circuitry. For example, hippocampal serotonin concentrations are elevated in male germ-free mice, but not females, compared with control mice with typical gut flora colonization (Clarke et al., 2013). However, immunological and neuroendocrine effects of changes to gut flora appear to be similar between males and females (Clarke et al., 2013). There has also been limited study on sex differences in epigenetic changes imposed by the early life environment. In humans, women have reduced global DNA methylation in peripheral blood compared with men, implying there may be differences in vulnerability to a challenge that influences methylation status (Zhang et al., 2011). However, methylation status of inflammatory markers such as IL-6 does not appear to be affected by sex (Zhang et al., 2012). Although there are sexually dimorphic effects of neonatal overfeeding on HPA axis responses to psychological stress (Spencer and Tilbrook, 2009), these do not seem to be apparent in the response to an immune challenge. As such, we have seen adult immune responses to LPS are exacerbated in both males and females made overweight due to neonatal overfeeding (Clarke et al., 2012). Thus, further work is necessary to clarify the differences, if there are any, between males and females in perinatal programming of neuroimmune function.

### Summary and future perspectives

Clearly, early life diet is essential for programming many aspects of adult physiology, including immune function and later susceptibility to disease. The gut microbiome and changes to the epigenetic profile may be particular mechanisms by which early life diet can alter immune function. In conjunction with these mechanisms, early life diet can predispose a subject to obesity, which has its own consequences for long-term immune function. Obesity that occurs as a result of early life diet may have independent implications for the immune system. Recent studies even imply that if one must become obese, there appear to be certain health advantages to doing it early on. At least, the basal pro-inflammatory profile, responses to a viral infection, and sickness behaviors seem to be unaffected in animals made obese by early life overfeeding, although febrile and cytokine responses to LPS are highly exacerbated. What this means for obese humans and for designing appropriate early life diets remains to be seen, but the implications for our immune systems are significant and clearly more work is needed in this field. Future research is needed to determine (1) how the early life gut microbiome can influence immune system development and if we can alter this with diet, (2) how early life influences, including diet, can cause epigenetic modifications to alter immune system development and if these can be reversed, and (3) how perinatal diet influences immune function independently of adult adiposity and if there is potential for early life interventions to reverse or ameliorate these effects.

## Acknowledgments and funding sources

This work was supported by a Discovery Project Grant from the Australian Research Council (ARC) to Sarah J. Spencer (DP109339), and Project Grant from the National Health and Medical Research Council (NHMRC) to Dr Zane Andrews and Sarah J. Spencer (APP1011274). Sarah J. Spencer is an ARC Future Fellow (FT110100084) and an RMIT University VC Senior Research Fellow.

### Conflict of interest statement

The author declares that the research was conducted in the absence of any commercial or financial relationships that could be construed as a potential conflict of interest.
